# Effects of various walking intensities on leg muscle fatigue and plantar pressure distributions

**DOI:** 10.1186/s12891-021-04705-8

**Published:** 2021-09-27

**Authors:** Chi-Wen Lung, Ben-Yi Liau, Joseph A. Peters, Li He, Runnell Townsend, Yih-Kuen Jan

**Affiliations:** 1 Rehabilitation Engineering Lab, Department of Kinesiology and Community Health, University of Illinois at Urbana-Champaign, Champaign, IL 61820 USA; 2 Department of Creative Product Design, Asia University, Taichung, 41354 Taiwan; 3 Department of Biomedical Engineering, Hungkuang University, Taichung, 433304 Taiwan; 4 College of Physical Education and Sports, Beijing Normal University, Beijing, 100875 China

**Keywords:** Complexity, Electromyography, Fatigue, Median frequency, Multiscale entropy, Peak plantar pressure

## Abstract

**Background:**

Physical activity may benefit health and reduce risk for chronic complications in normal and people with diabetes and peripheral vascular diseases. However, it is unclear whether leg muscle fatigue after weight-bearing physical activities, such as brisk walking, may increase risk for plantar tissue injury. In the literature, there is no evidence on the effect of muscle fatigue on plantar pressure after various walking intensities. The objectives of this study were to investigate the effects of various walking intensities on leg muscle fatigue and plantar pressure patterns.

**Methods:**

A 3 × 2 factorial design, including 3 walking speeds (1.8 (slow and normal walking), 3.6 (brisk walking), and 5.4 (slow running) mph) and 2 walking durations (10 and 20 min) for a total of 6 walking intensities, was tested in 12 healthy participants in 3 consecutive weeks. The median frequency and complexity of electromyographic (EMG) signals of tibialis anterior (TA) and gastrocnemius medialis (GM) were used to quantify muscle fatigue. Fourier transform was used to compute the median frequency and multiscale entropy was used to calculate complexity of EMG signals. Peak plantar pressure (PPP) values at the 4 plantar regions (big toe, first metatarsal head, second metatarsal head, and heel) were calculated.

**Results:**

Two-way ANOVA showed that the walking speed (at 1.8, 3.6, 5.4 mph) significantly affected leg muscle fatigue, and the duration factor (at 10 and 20 min) did not. The one-way ANOVA showed that there were four significant pairwise differences of the median frequency of TA, including walking speed of 1.8 and 3.6 mph (185.7 ± 6.1 vs. 164.9 ± 3.0 Hz, *P* = 0.006) and 1.8 and 5.4 mph (185.7 ± 6.1 vs. 164.5 ± 5.5 Hz, *P* = 0.006) for the 10-min duration; and walking speed of 1.8 and 3.6 mph (180.0 ± 5.9 vs. 163.1 ± 4.4 Hz, *P* = 0.024) and 1.8 and 5.4 mph (180.0 ± 5.9 vs. 162.8 ± 4.9 Hz, *P* = 0.023) for the 20-min duration. The complexity of TA showed a similar trend with the median frequency of TA. The median frequency of TA has a significant negative correlation with PPP on the big toe ( *r* = -0.954, *P* = 0.003) and the first metatarsal head ( *r* = -0.896, *P* = 0.016).

**Conclusions:**

This study demonstrated that brisk walking and slow running speeds (3.6 and 5.4 mph) cause an increase in muscle fatigue of TA compared to slow walking speed (1.8 mph); and the increased muscle fatigue is significantly related to a higher PPP.

## Background

Physical activity may benefit health and reduce risk of chronic complications in normal people and people with diabetes mellitus (DM) and peripheral vascular diseases [1]. The American Diabetes Association (ADA) recommends that people with DM should perform moderate-intensity aerobic exercise for 150 min/week or vigorous aerobic exercise for 75 min/week [2]. Moderate- and vigorous intensity exercise has been demonstrated to improve peripheral circulation, but light intensity exercise cannot [1]. Walking is the most common exercise in normal people and people with DM [3, 4]. However, walking is a weight-bearing exercise and fast walking such as brisk walking may cause high plantar pressure for increasing risk of plantar tissue injury and stress fracture of metatarsals [1, 3, 5–7]. Previous studies have confirmed that prolonged physical pressure over the skin is a major contributing factor for developing plantar tissue injury [5, 6].

Research studies have shown that muscle fatigue after intensive walking exercise reduces the body’s ability to absorb impact forces during walking [7, 8]. The reduced shock absorption often causes muscular and joint pain of the lower extremity and fall injury [7, 8]. Researchers usually focus on the postural adjustment and balance after leg muscle fatigue because of severe impact of fall injury [9]. In these studies, changes of plantar pressure, plantar tissue injury and metatarsal stress fracture are not well investigated [9]. In the literature, there are only few studies investigating changes of plantar pressures after various physical activities [7, 10, 11]. Ferris et al. found that plantar pressure values were affected by activities of lower extremity muscles [10]. Bisiaux and Moretto demonstrated that muscle fatigue of the lower extremity caused increased plantar pressure under the forefoot [11]. However, to the best of our knowledge, there is no study comparing changes of plantar pressure after various walking intensities and relationship between leg muscle fatigue and plantar pressure patterns. Although moderate intensity exercise, such as brisk walking is recommended for the normal people and people with DM, it is unclear whether these moderate and vigorous exercise are associated with increased leg muscle fatigue and increased plantar pressures as well as risk for plantar tissue injury. Because various healthcare clinicians have been strongly advocating physical activity in people with DM and peripheral vascular diseases, there is an increasing need to understand the relationship between muscle fatigue and plantar pressure changes.

The objectives of this study were to investigate the effects of different walking intensities on leg muscle fatigue and plantar pressure as well as to investigate the relationship between leg muscle fatigue and plantar pressure. To the best of our knowledge, this is the first study to compare leg muscle fatigue and plantar pressure patterns after various walking speeds and durations. The results from this study in healthy participants can provide a foundation for understanding the effect of walking intensities on leg muscle fatigue and plantar pressure in people with DM and peripheral vascular diseases. We hypothesized that different walking speeds (ranging from slow walking, brisk walking to slow running) and durations cause different degrees of leg muscle fatigue and plantar pressure patterns.

## Methods

A 3 × 2 factorial design, including three speeds (1.8, 3.6, and 5.4 mph) and two durations (10 and 20 min), was used in the current study. This study was part of a larger project investigating the effect of walking intensities on plantar skin blood flow responses [3, 4]. The EMG data has not been reported elsewhere. These three walking speeds represent slow and normal walking at 1.8 mph, brisk walking at 3.6 mph, and slow running at 5.4 mph. We also intended to examine the differences between two common walking durations of 10 and 20 min [3, 4]. The specific walking tasks were as follows.

- Walking at 1.8 mph for 10 min in the first week
- Walking at 1.8 mph for 20 min in the first week
- Walking at 3.6 mph for 10 min in the second week
- Walking at 3.6 mph for 20 min in the second week
- Walking at 5.4 mph for 10 min in the third week
- Walking at 5.4 mph for 20 min in the third week

### Participants

Healthy participants between 20 and 45 years of age were recruited from the university and nearby community. Exclusion criteria were active foot ulcers, diabetes, vascular diseases, hypertension, the inability to walk for 20-min independently, the inability to walk at the speed of 5.4 mph independently, or the use of vasoactive medications [3, 4]. The examinations were performed at the Rehabilitation Engineering Laboratory, University of Illinois at Urbana-Champaign with room temperature at 24 ± 2 °C. The study was approved by the Institutional Review Board, University of Illinois at Urbana-Champaign (IRB #19,225). Each participant signed the informed consent for this study.

### Experimental procedures

The research participant took off the socks and shoes and was in the supine position for 30 min before the walking protocol to avoid the influence of previous weight-bearing activities on muscle fatigue and plantar pressure. All participants were asked to walk with an appropriate size of standard shoes (1-inch heel, Altrex, Teaneck, NJ, USA) at a speed of 1.8 mph on a treadmill in the first visit. Electromyographic (EMG) electrodes (Model EL507 (Bipolar Ag/AgCl electrodes) and EMG100C, Biopac Systems, Inc., Goleta, CA, USA) were taped on the tibialis anterior (TA) and gastrocnemius medialis (GM) of the dominant leg according to SENIAM locations [12]. The sampling rate of EMG signals was 1,000 Hz. The skin sites for placing the electrodes were cleaned using alcohol wipes and shaved. The plantar pressure insole measure system was used to measure plantar pressures (Tekscan, South Boston, MA, USA). The participant wore the insole sensors inside the shoe for 3-min walking before the calibration. The insole sensor was then calibrated according to the manufacturer’s guidelines [13, 14]. Plantar pressure data were collected at 300 Hz. Participants were randomly assigned into the 10- or 20-min walking duration first and the other duration later, with a balanced crossover design. The random order of 10- and 20-min duration minimized the order effect of duration factor on EMG and plantar pressure responses. For avoiding carryover effects on muscle fatigue and plantar pressure, participants were allowed to rest for at least 20 min between 10- and 20-min walking trials. All participants returned to the laboratory for performing 3.6 mph in the second visit and 5.4 mph walking in the third visit. Each visit was separated between 7 ± 2 days.

### EMG and plantar pressure analyses

**Fig. 1:**
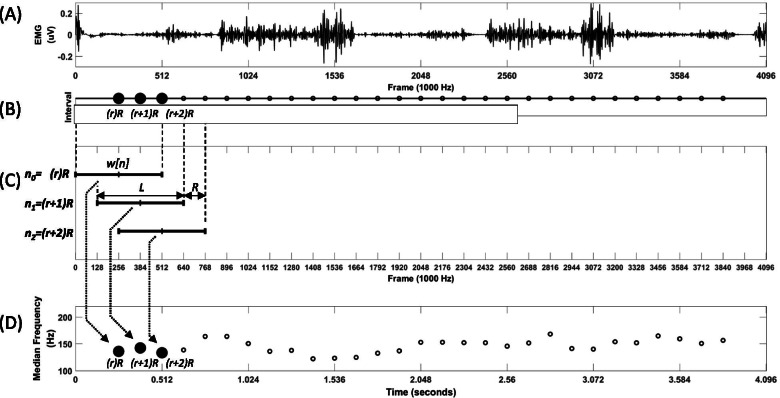
The short-time Fourier transform for muscle fatigue quantification during walking. **A** The raw data of EMG signal (**A**) is filtered by a band pass filter as the filtered signal (**B**). **C** The signal was extracted using the window sequence *w[n]* of length *L*, with shift *(r)* of *R* samples. **D** Estimation of the median frequency using the windowed sequence, and the average values of the median frequency were calculated under 6 walking conditions

The EMG data were analyzed using the median frequency and multiscale entropy (MSE) methods to characterize muscle fatigue at different walking intensities. The EMG signals were filtered using a zero-lag band pass filter (Butterworth filter, 6^th^ order, 5–400 Hz) [15]. The power spectrum median frequency of the EMG was determined by short-time Fourier transforms. Each of the short-time segments was multiplied by the Hamming window. The window size was 512 ms. The sampling interval in the time domain was 75% of the window length. The average value of median frequency in each short-time segment was used as a fatigue index of muscles in each walking condition. EMG data were processed using Matlab 2020b (The MathWorks, Natick, MA, USA) (Fig. 1). The median frequency of EMG signals was used to detect muscle fatigue. When muscle becomes fatigue, the median frequency of EMG spectrum shifts to a lower value. Muscle fatigue is related to insufficient blood perfusion of muscle fibers, depletion of energy sources, and metabolites' build-up. The build-up of excess hydrogen ions slows down the waveform of the action potential in muscle contraction, resulting in two electrophysiological events. The build-up of excess hydrogen ions slows down the waveform of an action potential in a muscle contraction, resulting in two electrophysiological events: Synchronization of the motor unit pool leads to an increase in amplitude and an increase in the duration of the activation of the EMG signal [16]. This could be used to explain the shift to a lower median frequency after muscle fatigue.

Multiscale entropy (MSE) was used to assess the complexity change of muscle fatigue. Recent studies have demonstrated that both linear methods (median frequency) and nonlinear methods (eg. entropy and fractal) should be used to characterize changes of EMG signals. The use of only one approach (either linear or nonlinear) will not be able to fully characterize neuromuscular changes of fatigued muscle [17]. MSE is one of the methods developed by Costa et al. [18–20]. MSE uses the “Sample Entropy” algorithm to estimate the regularity in different time scale based on the approximate entropy to assess the complexity degree. MSE has been used to investigate pathological changes of neuromuscular regulations in patients with neurological impairments and is able to detect a decreasing pattern of EMG complexity [17, 21]. Although the exact physiopathological meanings of EMG complexity are still largely unknown, these studies speculated that the EMG complexity reflects the complex motor unit recruitment and synchronization issues of neuromuscular system that cannot be detected by traditional time and frequency domain analyses [21, 22].

First, a one-dimensional discrete time series [× 1, × 2, …. xn] is reconstructed by the scale factor “τ” to be coarse-grained time series with a different time scale. Each element of ${\mathrm{y}}_{\mathrm{j}}^{(\uptau )}$ is according to Eq. (1)

1 $${\mathrm y}_{\mathrm j} ^ {(\tau )} = {\frac{1} {\mathrm\tau}} {\sum}_{\mathrm i = \left(\mathrm j-1\right) {\mathrm\tau} +1} ^{\mathrm j {\mathrm\tau}} {\mathrm x}_{\mathrm i},1\le \mathrm j \le \frac{\mathrm N} {\mathrm\tau}$$

By the coarse-grained procedure, Sample Entropy (SE) could be estimated by the scale factor “τ.” SE is defined by Eq. (2)

Where “m” is the template vector of length,

“A” is the number of template vector pairs having $\mathrm{d}[{\mathrm{x}}_{\mathrm{m}+1}\left(\mathrm{i}\right),{\mathrm{x}}_{\mathrm{m}+1}(\mathrm{j})]<\gamma$

“B” is the number of template vector pairs having $\mathrm{d}[{\mathrm{x}}_{\mathrm{m}}\left(\mathrm{i}\right),{\mathrm{x}}_{\mathrm{m}}(\mathrm{j})]<\gamma$

2 $$\mathrm{SE}\left(\mathrm{m},\upgamma ,\uptau \right)=-\mathrm{log}\frac{{\mathrm{A}}_{\uptau }}{{\mathrm{B}}_{\uptau }}$$

Complexity index (CI) could be estimated from SE by Eq. (3), which is the summation of SE from scale factor 1 to maximum.

3 $$\mathrm{CI}={\sum }_{\mathrm{i}=1}^{\uptau }\mathrm{SE}(\mathrm{i})$$

Plantar pressures were se averaged to determine the four plantar regions at high risk of diabetic foot ulcers [23]. The four plantar foot regions were the 1st toe (T1), 1st metatarsal head (M1), 2nd metatarsal head (M2), and heel (HL). Data from the three intermediate steps of the last-minute (i.e., 9^th^ min and 19^th^ min) of the treadmill walking were processed to calculate the average peak plantar pressure (PPP) across the three steps [13, 14].

### Statistical analysis

The median frequency and MSE values of EMG of TA and GM and PPP of each participant at 6 walking protocols (ie. three speeds (slow at 1.8 mph, moderate at 3.6 mph, and fast at 5.4 mph) and two durations (10 and 20 min)) were presented as mean ± standard error. The 3 × 2 two-way analysis of variance (ANOVA) with repeated measures was used to compare the muscle fatigue changes between the three speeds (1.8, 3.6, and 5.4 mph) and two durations (10 and 20 min) and the interaction between walking speeds and durations on muscle fatigue. One-way analysis of variance (ANOVA) with Fisher’s least significant difference (LSD) post hoc tests were used to compare pairwise median frequency differentials between 1.8 mph, 3.6 mph, and 5.4 mph for the two walking durations. The correlations between the median frequency and MSE of TA and GM and PPP of four plantar regions were determined using a Pearson product-moment correlation analysis. All statistical tests were performed using SPSS 25 (IBM, NY, USA) at the significance level of 0.05.

## Results

Twelve healthy participants (5 men, 7 women) were recruited in this study. The demographic data were (mean ± standard deviation): age, 27.1 ± 5.8 years; height, 170.3 ± 10.0 cm; and weight, 63.5 ± 13.5 kg. The dominating leg of all subjects is the right side.

### Median frequency of muscle fatigue

**Fig. 2:**
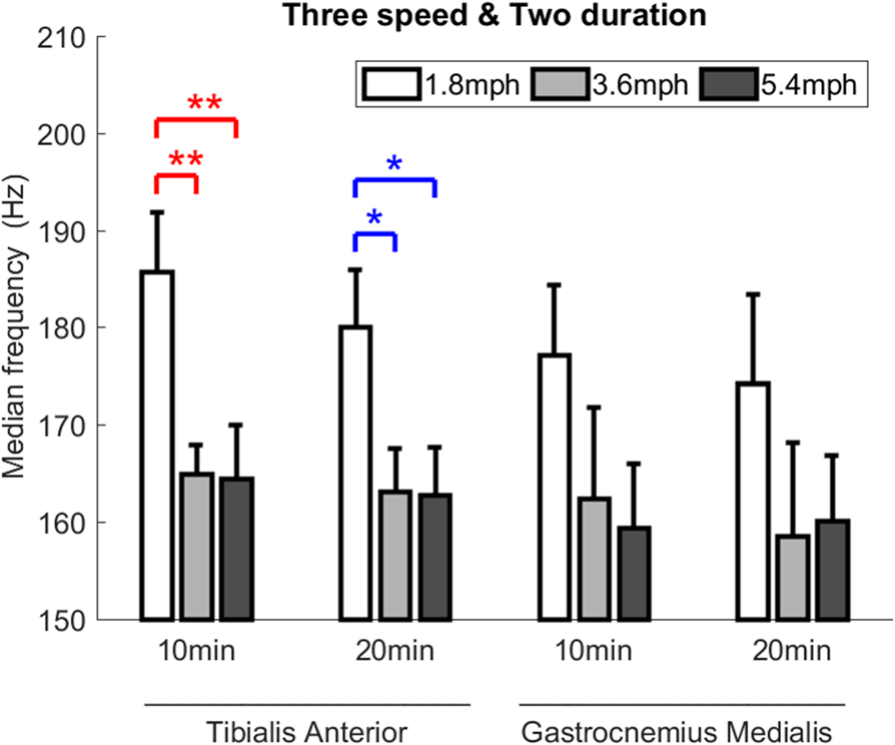
Comparisons of EMG median frequency of the tibialis anterior and gastrocnemius medialis after 6 walking intensities. Data are shown as mean ± standard errors. *P* < 0.05 (*) and *P* < 0.01 (**)

The 3 × 2 two-way ANOVA (3 speeds and 2 durations) showed that the speed factor caused a significant main effect of the median frequency of muscle fatigue of TA (*P* < 0.001) and the duration factor did not significantly change the median frequency. There was no interaction between the speed and duration factors on median frequency of TA and GM (Fig. 2 and Table 1).

**Table 1:** The median frequency and MSE of EMG of tibialis anterior (TA) and gastrocnemius (GM), and peak plantar pressure (PPP) of four plantar regions

**Parameter**	**Region**	**Speed & Duration**					
		**1.8 mph &**	**1.8 mph &**	**3.6 mph &**	**3.6 mph &**	**5.4 mph &**	**5.4 mph &**
		**10-min** (Mean ± SE)	**20-min** (Mean ± SE)	**10-min** (Mean ± SE)	**20-min** (Mean ± SE)	**10-min** (Mean ± SE)	**20-min** (Mean ± SE)
Median frequency (Hz)	TA	185.7 ± 6.1	179.9 ± 5.8	165.0 ± 3.1	163.0 ± 4.4	164.6 ± 5.5	162.6 ± 4.9
	GM	177.0 ± 7.3	174.2 ± 9.2	162.4 ± 9.4	158.5 ± 9.5	159.5 ± 6.6	160.0 ± 6.7
MSE (CI)	TA	0.886 ± 0.030	0.897 ± 0.041	0.840 ± 0.0450.039	0.858 ± 0.040	0.989 ± 0.045	0.939 ± 0.041
	GM	0.662 ± 0.045	0.642 ± 0.036	0.703 ± 0.0410.026	0.758 ± 0.0360.045	0.644 ± 0.032	0.622 ± 0.030
PPP (kPa)	T1	275.4 ± 48.7	333.8 ± 64.9	460.8 ± 66.5	456.0 ± 87.9	509.1 ± 90.7	548.7 ± 77.4
	M1	369.4 ± 59.3	408.3 ± 70.2	462.4 ± 64.4	575.5 ± 109.9	591.4 ± 87.3	601.4 ± 77.9
	M2	587.0 ± 67.0	583.6 ± 57.0	567.4 ± 75.4	606.8 ± 83.4	613.0 ± 84.1	719.2 ± 79.4
	HL	313.5 ± 41.9	350.0 ± 43.0	491.4 ± 53.9	527.9 ± 81.6	403.8 ± 63.4	410.7 ± 58.1

*TA* Tibialis anterior, *GM* Gastrocnemius medialis, *MSE* Multiscale entropy, *CI* Complexity index, *T1* 1^st^ toe, *M1* 1^st^ metatarsal head, *M2* 2^nd^ metatarsal head, and *HL* heel, *SE* Standard error

For the EMG median frequency of TA, the one-way ANOVA showed that there were four significant pairwise differences, including the between walking speed of 1.8 and 3.6 mph (185.7 ± 6.1 vs. 164.9 ± 3.0 Hz, *P* = 0.006) and 1.8 and 5.4 mph (185.7 ± 6.1 vs. 164.5 ± 5.5 Hz, *P* = 0.006) for the 10-min duration; and between walking speed of 1.8 and 3.6 mph (180.0 ± 5.9 vs. 163.1 ± 4.4 Hz, *P* = 0.024) and 1.8 and 5.4 mph (180.0 ± 5.9 vs. 162.8 ± 4.9 Hz, *P* = 0.023) for the 20-min duration (Fig. 2 and Table 1).

### Complexity index of muscle fatigue

**Fig. 3:**
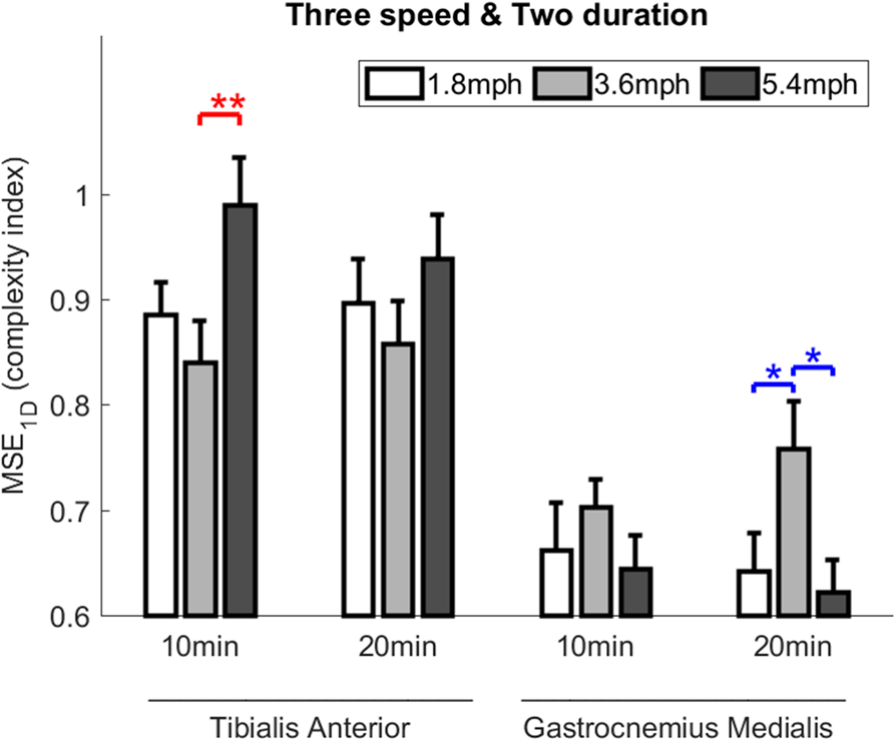
Comparisons of EMG complexity of the tibialis anterior and gastrocnemius medialis after 6 walking intensities. Data are shown as mean ± standard errors. Complexity is calculated by multiscale entropy (MSE). *P* < 0.05 (*) and *P* < 0.01 (**)

The 3 × 2 two-way ANOVA (3 speeds and 2 durations) showed that the speed factor caused a significant main effect of the complexity index of TA (*P* = 0.017) and GM (*P* = 0.023) and the duration factor did not. There was no interaction between the speed and duration factors on the complexity of TA and GM (Fig. 3 and Table 1).

The one-way ANOVA showed three significant pairwise differences in the effect of walking speeds on the EMG complexity index. TA showed that between the walking speed of 3.6 and 5.4 mph (0.840 ± 0.039 vs. 0.989 ± 0.045, *P* = 0.010) during the 20-min duration. GM showed that between the walking speed of 1.8 and 3.6 mph (0.758 ± 0.045 vs. 0.642 ± 0.036, *P* = 0.037) in the 20-min duration (Fig. 3 and Table 1).

### Correlations between the median frequency and complexity of EMG and PPP

**Fig. 4:**
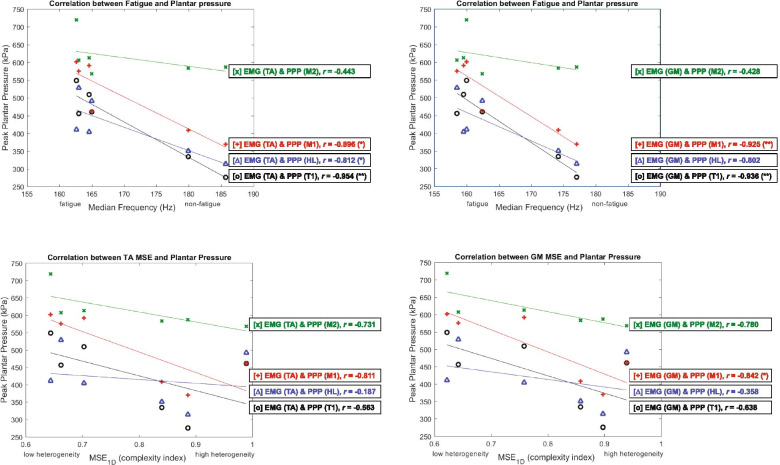
The scatter plots showing the relationship between the median frequency and MSE of EMG signals of tibialis anterior (TA) and gastrocnemius medialis (GM) and PPP at four plantar foot regions. **A** Median frequency of TA and PPP. **B** Median frequency of GM and PPP. **C** Complexity of TA (calculated by multiscale entropy (MSE)) and PPP. **D** Complexity of GM and PPP. Notes: 1^st^ toe (T1); 1^st^ metatarsal head (M1); 2^nd^ metatarsal head (M2); heel (HL); *P* < 0.05 (*); *P* < 0.01 (**). Fatigue and non-fatigue conditions are relative in this study based on the shift to lower median frequency in the speeds of 3.6 and 5.4 mph compared to 1.8 mph

In the correlation between the EMG median frequency and PPP, the median frequency of TA has a significant negative correlation with PPP on T1 ( *r* = -0.954, *P* = 0.003), M1 ( *r* = -0.896, *P* = 0.016), and HL (*r* = -0.812, *P* = 0.050). The median frequency of GM has a significant negative correlation with PPP on T1 (*r* = -0.939, *P* = 0.006) and M1 ( *r* = -0.925, *P* = 0.008) (Fig. 4 and Table 2).

**Table 2:** The correlations between the median frequency and MSE of EMG of tibialis anterior (TA) and gastrocnemius medialis (GM) with the peak plantar pressure (PPP)

Parameter	Region	EMG of Median frequency				EMG of MSE			
		TA		GM		TA		GM	
		Correlation	*P* value	Correlation	*P* value	Correlation	*P* value	Correlation	*P* value
PPP	T1	-0.954	0.003**	-0.939	0.006**	-0.563	0.245	-0.638	0.172
	M1	-0.896	0.016*	-0.925	0.008**	-0.811	0.050	-0.842	0.036*
	M2	-0.443	0.379	-0.428	0.397	-0.731	0.099	-0.780	0.067
	HL	-0.812	0.050*	-0.802	0.055	-0.187	0.722	-0.358	0.486

*MSE* Multiscale entropy, *CI* Complexity index, *T1* 1^st^ toe, *M1* 1^st^ metatarsal head, *M2* 2^nd^ metatarsal head, and *HL* heel, *SE* Standard error

*P* < 0.05 (*) and *P* < 0.01 (**)

In the correlation between the EMG complexity index and PPP, GM has a significant negative correlation with PPP on M1 ( *r* = -0.842, *P* = 0.036) (Fig. 4 and Table 2).

## Discussion

The results of this study demonstrated that the walking speed (1.8, 3.6, and 5.4 mph) is a significant factor on inducing leg muscle fatigue and the walking duration (10 and 20 min) is not. Specifically, the median frequency of TA EMG significantly decreased after brisk walking (3.6 mph) and slow running (5.4 mph) compared to slow walking (1.8 mph). Although the median frequency of GM EMG showed a similar trend to TA EMG, the median frequency of GM EMG did not significantly change. The multiscale entropy based complexity of TA EMG showed a similar trend to the median frequency. Another significant finding is that muscle fatigue assessed by the median frequency and complexity were significantly correlated to the plantar pressure changes after walking at various intensities.

Our results showed that under 6 walking intensities, TA was significantly fatigued, but GM was not. This is consistent with the literature that TA may become fatigued earlier compared to GM [7, 24]. Blair et al. proposed that compared to GM muscle, TA muscle activity increased significantly during raising the walking speed [24]. During the early swing, the TA actively shortens for ankle dorsiflexion and for clearing the toes from the ground. However, the majority of the shortening of TA occurs through tendinous tissue recoil at the fast walking speed, highlighting its role in accelerating ankle dorsiflexion [25]. Our results imply that walking speed at 3.6 mph (brisk walking speed) and 5.4 mph (slow running) may easily cause TA fatigue. Thus, people with DM should be reminded that when they pursing moderate-to-vigorous physical activity, muscle fatigue of lower extremity may occur and result in higher plantar pressure that may increase risk for DFUs [13, 14]. Our finding also suggests that slow walking speed (1.8 mph) may be a good strategy for preventing muscle fatigue; therefore, preventing high plantar pressure during weight-bearing physical activities. We also found that the GM may not become fatigued easily compared to TA. This implies that in order to prevent muscle-fatigue related increases in plantar pressure, TA should be the main muscle for rehabilitation and exercise training in people at risk for foot injury.

In this study, we observed that muscle fatigue is related to a higher plantar pressure. The reason is that muscle fatigue may decrease the muscle contraction force and induce the biomechanical changes for increasing plantar pressure. Garrett et al. found that the energy absorption was higher for the contracted muscles than the uncontracted muscles in an experimental animal model [26]. When muscle is fatigue, muscle may become stiffer and may not effectively respond to neuromuscular control to shorten during muscle contractions [27]. A decrease in muscle force production during fatigue may reduce impact force absorption and increase plantar pressure [28]. Our study confirms that the increased plantar pressure was correlated with muscle fatigue after various walking intensities. EMG signals are typically not directly proportional to the muscle contraction force due to the other component effects. However, EMG has been shown to effectively characterize muscle fatigue process [29]. However, the relationship between muscle fatigue and increased plantar pressure observed in this study requires experimental studies to validate.

Previous investigations of the unfatigued state showed that initial heel contact causes a plantarflexion moment. The heel is slightly supinated throughout touchdown, pronates during the stance phase of walking, and returns to supination at push-off of the walking stage [30]. In the fatigued state locomotion, people use a change in the landing technique as a compensatory strategy, which may cause an external dorsiflexion moment [31]. This adaptation change in forefoot loading has been suggested as a potential mechanism for increased plantar pressure [32]. According to the present findings, the forefoot loading point toward a potentially detrimental plantar pressure mechanism in fatigued walking decreases calf muscle activity. This may reflect that the 2^nd^ metatarsal head has a higher plantar pressure.

EMG usually could be analyzed by the linear and nonlinear approaches. However, some information and message were hidden by those approaches. MSE analysis is a practical nonlinear approach to provide more information insight about the results due to its pre-processing of various time scales in the algorithm. Different walking intensities induce variability of muscle activation [33]. Our results indicated that the EMG analysis by MSE in the moderate walking speed, the GM muscle revealed a higher complexity index, and the TA muscle revealed a lower complexity index. Although the lower complexity index in TA muscle activation may induce another variability pattern for GM muscle activation, GM and TA muscle may exhibit compensation by their different functional performance during walking in the moderate walking speed. Furthermore, the duration of exercise would induce differences in complexity indexes in muscle activations. Our findings indicate that the EMG complexity index might reveal differences in TA with 10-min walking duration and GM muscle with 20-min walking duration. It supports the previous report that a more significant proportion of the variability in performance on time variability for walking tests [34].

Our finding showing that muscle fatigue may be related to a higher plantar pressure has significant clinical implications on managing risk of plantar tissue injury and metatarsal stress fracture. When a person with peripheral vascular diseases or diabetes pursues an intensive exercise, muscle fatigue of lower extremity may be expected. These fatigued muscles may result in a higher plantar pressure that increases the risk for plantar tissue injury [13, 14]. In the clinical guidelines, moderate-intensity exercise such as slow running (5.4 mph) is recommended for people with peripheral vascular disease. These people should pay attention to their plantar foot after exercise, especially after brisk walking and slow running activities. Clinicians and sports coaches should be aware that moderate and vigorous intensity exercise may provide more benefit to improve cardiopulmonary functions, but at the same time, these intensive exercise may increase risk for plantar tissue injury and metatarsal stress fracture, especially in people with DM and peripheral vascular diseases.

There are limitations in this study. First, we only assessed muscle fatigue of TA and GM through the median frequency and complexity of EMG and peak plantar pressures after walking at various intensities. However, various biomechanical characteristics of human walking, such as joint kinematics, kinetics, and muscle activities [24, 35, 36], were not assessed. Future research may need to assess these biomechanical parameters to better understand how various walking intensities affect muscle fatigue and plantar pressures. Second, the walking duration tested in this study (10 and 20 min) was relatively short compared to activities of daily living. Future studies may need to assess a longer walking duration, such as 60 min. Third, the washout period between 20 and 30 min was allowed between the two walking durations of the same speed of a day to minimize the influence of muscle fatigue. Although the participants felt comfortable before participating in the second walking protocol of a day, the influence of muscle fatigue from the first walking protocol may affect the second walking protocol of a day. Last, we assessed EMG and plantar pressure responses in 6 walking trials in three consecutive weeks. The temporal variations of median frequencies of EMG signals and plantar pressure may affect our findings. Researchers may use established fatigue protocols to examine the effect of leg muscle fatigue on plantar pressure.

## Conclusions

The results of this study demonstrate that the walking speed (1.8, 3.6, and 5.4 mph) is a significant factor on inducing leg muscle fatigue and the walking duration (10 and 20 min) is not. The median frequency of tibialis anterior significantly decreased after brisk walking (3.6 mph) and slow running (5.4 mph) speed compared to slow walking (1.8 mph) speed. The multiscale entropy based complexity of TA EMG showed a similar trend to the median frequency. Another significant finding is that muscle fatigue assessed by the median frequency and complexity were significantly correlated to the plantar pressure changes after walking at various intensities.

## Data Availability

The datasets generated and analyzed during the current study are not publicly available due to an ongoing computational modeling project but are available from the corresponding author on reasonable request.

## Abbreviations

ANOVA: Analysis of variance
CI: Complexity index
DFU: Diabetic foot ulcers
DM: Diabetes mellitus
EMG: Electromyography
GM: Gastrocnemius medialis
LSD: Least significant difference
TA: Tibialis anterior
